# Load Balancing Using Artificial Intelligence for Cloud-Enabled Internet of Everything in Healthcare Domain

**DOI:** 10.3390/s23115349

**Published:** 2023-06-05

**Authors:** Ibrahim Aqeel, Ibrahim Mohsen Khormi, Surbhi Bhatia Khan, Mohammed Shuaib, Ahlam Almusharraf, Shadab Alam, Nora A. Alkhaldi

**Affiliations:** 1 College of Computer Science & IT, Jazan University, Jazan 45142, Saudi Arabia; iahmed@jazanu.edu.sa (I.A.); ikhormi@jazanu.edu.sa (I.M.K.); talkshuaib@gmail.com (M.S.); 2 Department of Electrical and Computer Engineering, Lebanese American University, Byblos 13-5053, Lebanon; 3 Department of Data Science, School of Science, Engineering and Environment, University of Salford, Manchester M5 4WT, UK; 4 Department of Business Administration, College of Business and Administration, Princess Nourah bint Abdulrahman University, P.O. Box 84428, Riyadh 11671, Saudi Arabia; aialmusharraf@pnu.edu.sa; 5 Department of Computer Science, College of Computer Science and Information Technology, King Faisal University, Al Hasa 31982, Saudi Arabia; nalkhaldi@kfu.edu.sa

**Keywords:** Internet of Things, Internet of Everything, big data analytics, cloud computing, clustering, load balancing, healthcare

## Abstract

The emergence of the Internet of Things (IoT) and its subsequent evolution into the Internet of Everything (IoE) is a result of the rapid growth of information and communication technologies (ICT). However, implementing these technologies comes with certain obstacles, such as the limited availability of energy resources and processing power. Consequently, there is a need for energy-efficient and intelligent load-balancing models, particularly in healthcare, where real-time applications generate large volumes of data. This paper proposes a novel, energy-aware artificial intelligence (AI)-based load balancing model that employs the Chaotic Horse Ride Optimization Algorithm (CHROA) and big data analytics (BDA) for cloud-enabled IoT environments. The CHROA technique enhances the optimization capacity of the Horse Ride Optimization Algorithm (HROA) using chaotic principles. The proposed CHROA model balances the load, optimizes available energy resources using AI techniques, and is evaluated using various metrics. Experimental results show that the CHROA model outperforms existing models. For instance, while the Artificial Bee Colony (ABC), Gravitational Search Algorithm (GSA), and Whale Defense Algorithm with Firefly Algorithm (WD-FA) techniques attain average throughputs of 58.247 Kbps, 59.957 Kbps, and 60.819 Kbps, respectively, the CHROA model achieves an average throughput of 70.122 Kbps. The proposed CHROA-based model presents an innovative approach to intelligent load balancing and energy optimization in cloud-enabled IoT environments. The results highlight its potential to address critical challenges and contribute to developing efficient and sustainable IoT/IoE solutions.

## 1. Introduction

**Figure 1:**
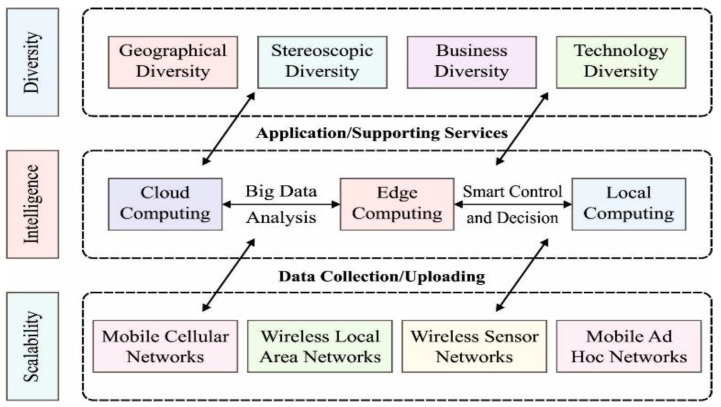
General expectation of IoE.

The concept of IoT is evolving, leading to the emergence of the broader subset known as IoE, which aims to connect devices, data, and industries. While IoT refers to the network of interconnected physical devices embedded with sensors and software, enabling them to collect and exchange data, IoE is a broader concept that extends beyond IoT by integrating people, processes, and data into a comprehensive, interconnected ecosystem [1]. IoE goes beyond connecting gadgets and includes people, data, and various sectors such as emergency response, urban planning, healthcare, and the military [2]. Data integration and contextualization are crucial in IoE due to the many components and data sources involved. A typical ecosystem is needed to enable communication and integration between information, systems, and sensor inputs. IoE nodes, such as tablets, smartphones, and home appliances, detect the environment, exchange data, and possess different capabilities [3]. The intelligence of IoE nodes lies in their ability to share and interact with data, learning from it to perform valuable activities [4]. The availability of IoT data and the development of AI contribute to the intelligence of IoE. IoE finds applications in intelligent transportation, smart agriculture systems, and smart manufacturing. The growth of ICT technology, from human-oriented Internet to machine-oriented IoT and advancements such as WSN, LPWAN, and 5G, enables the realization of IoE and facilitates machine-to-machine communication [5]. A general expectation of IoE is presented in Figure 1.

The rapid growth of the Internet of Things (IoT) and advancements in artificial intelligence have enabled intelligent applications in various fields such as healthcare, smart cities, and Industry 4.0. These applications improve decision-making, recognition, and management processes [6]. The IoT has evolved into the Internet of Everything (IoE), connecting and enabling intelligent uses such as smart metering, traffic scheduling, and agriculture. Wireless transmission networks such as 5G, WSNs, and LPWAN contribute to the scalability of the IoE. At the same time, big data analysis is crucial for extracting valuable insights from data for marketing, analytics, and machine learning purposes [7,8]. Data extraction is essential in ingesting data for analysis and decision-making [2]. Artificial intelligence further enhances the intelligence of the IoE.

Despite the advancements, there are inherent constraints in the IoE, including battery limitations of the IoT nodes, network coverage limitations, and security vulnerabilities [9,10].

These constraints affect the availability and connectivity of IoT devices, especially in coverage-limited areas. Clustering techniques group similar objects, enabling valuable insights and efficient system performance. However, scalability issues can arise when IoT devices struggle to adapt to platform changes and fail to meet client expectations [11].

A novel energy-aware cluster-based load balancing approach called CHROA is proposed to address these challenges for a cloud-enabled IoT environment. The CHROA model utilizes clustering to effectively balance the load and optimize energy resources. It incorporates the concept of chaos into the HROA technique to enhance global optimization capabilities. The CHROA technique is implemented using Apache Flume, a Big Data platform, and the Hadoop Distributed File System (HDFS) for storage. The method considers input variables such as energy, distance, and delay factor to build an objective function. The performance of the CHROA method is validated using a MATLAB program and evaluated in various settings.

### 1.1. Importance of Load Balancing in Internet of Everything

Load balancing is of utmost importance in the Internet of Everything (IoE) for various reasons. Firstly, it significantly enhances system performance by evenly distributing network traffic among multiple devices. This prevents congestion and ensures that essential applications and services in healthcare, for instance, are readily accessible to users whenever they need them. Secondly, load balancing enhances system reliability by eliminating the risks associated with single points of failure. In the event of a device failure, traffic can be seamlessly redirected to alternative devices, thereby ensuring uninterrupted service delivery. Thirdly, load balancing plays a critical role in bolstering security measures [12]. By dispersing traffic across numerous devices, it becomes considerably more challenging for potential attackers to target a single device, effectively reducing the risk of cyberattacks and safeguarding sensitive data [13]. Moreover, load balancing optimizes resource utilization by efficiently managing traffic distribution, preventing individual devices from being overwhelmed. This leads to cost reductions and improved scalability, making it easier to accommodate increasing demands. Overall, load balancing is an indispensable component of the IoE as it improves performance, increases reliability, enhances security, and optimizes resource utilization, ultimately enabling the delivery of top-quality services across a diverse range of industries.

### 1.2. IoT, IoE, and Role of Load Balancing in Healthcare

The Internet of Things (IoT) and the Internet of Everything (IoE) are distinct concepts, with the IoE encompassing a broader scope that includes people, processes, and data. In healthcare, both IoT and IoE have transformative potential [14]. IoT devices such as wearables and sensors collect real-time patient health data, enabling monitoring and improved treatment outcomes [15]. IoE integrates data analytics, AI, and other technologies to view patient health comprehensively. By combining various data sources, including electronic health records and environmental factors, IoE enhances healthcare decision-making. Load balancing plays a crucial role in healthcare by evenly distributing network traffic across devices, improving system performance, and ensuring timely access to healthcare applications [16]. It also increases system reliability by eliminating single points of failure and enhances security by dispersing traffic to reduce the risk of cyberattacks. Moreover, load balancing optimizes resource utilization, avoiding device overloads, reducing costs, and improving scalability. Overall, IoT and IoE have the potential to revolutionize healthcare, improving patient outcomes, increasing efficiency, and reducing costs.

This research seeks to develop intelligent and energy-efficient load-balancing models for cloud-enabled IoT in healthcare. By leveraging AI and big data analytics, the proposed model seeks to optimize resource utilization, enhance performance, and improve the delivery of healthcare services, thereby contributing to efficient and sustainable IoE solutions and ultimately improving patient outcomes.

The distinctive contributions of this paper are:

1. Building a low-power data retrieval system for the IoE.
2. The proposed optimization algorithm can be adapted for use in the IoE to facilitate the formation of clusters of related sensors.
3. Application of CHROA to elect optimal cluster heads (CHs).
4. To optimize the energy efficiency and cost-effectiveness of the Fog-IoE framework and create a method for handling end-user requests.
5. Improve resource utilization and reduce the need to move tasks to the cloud by proposing a load-balancing strategy for fog computing.

The following layout depicts the organizational structure for the rest of this project. Section 2 provides a more comprehensive review of the IoE-related works. The proposed framework will be made public in the third step, and the simulation process will be evaluated in the fourth. The conclusion is found in the fourth and final portions.

## 2. Literature Review

A brief review of related works has been completed related to the IoE. Cao et al. [17] developed the Edge OSH concept, a home operating system for the IoE. They also covered functional issues such as self-management, encoding interface, security and privacy, naming, and data management, as well as non-functional issues such as lack of open testbed availability, latency, system cost, and user experience. Naranjo et al. [18] presented a Fog-based smart city network named FOCAN, a multi-tier architecture where the application runs on things that mutually route, compute, and interact via a smart city platform. The FOCAN reduces delay and enhances the energy provision and the efficacy of services between things using distinct abilities. Mainly, three kinds of communications are determined with FOCAN devices, primary, secondary, inter-primary and transmission, for managing applications that meet the standard quality of service (QoS) for the IoE.

Singh et al. [19] drew inspiration from Blockchain and FC approaches to create the BFAN-safe architecture. The provided structure uses Blockchain, encryption, and authentication to safeguard sensitive data. It facilitates deploying applications in smart city paradigms for architects and system developers. The presented framework aims to reduce the delay and guarantee an enhanced security feature via a blockchain technique. Divyabharathi et al. [20] proposed the first-ever Blockchain that could instantaneously manage the data security and device that is significant to emerge in the IoE. It introduces an exclusive idea of Blockchain, which incorporates hardware security primitives named PUF for solving latency, scalability, and energy need problems, called a PUF chain. Miao et al. [21] offered a fair and dynamic data-sharing framework (FairDynDSF) in a multi-owner setup. The accuracy of the search results must be validated, perform multi-keyword searches, dynamic upgrades, and achieve fair arbitration. In FCN, Xiao et al. [22] applied the time sharing (TS) strategy in place of the first come, first served (FCFS).

FCN is next supplied with the collaborative LB methodology for the TS method. The approach differs from a work theft scheduling method in that it is based on the Nash bargaining solution (NBS) for distributed games among FCNs. FCNs work together to improve the efficiency of all FCNs to achieve Pareto optimality. Chithambaramani et al. [23] examined the computational offloading problems of the existence and synergy between FC and CC in the IoE by mutually improving the offloading decision, the distribution of computational assets, and transfer power. Primarily, they proposed an ECORA system to minimize the scheme cost. Garzia and Papi [24] aimed to tell how an IoE-based unified security system for archaeology areas may assure cultural heritage preservation or conservation, outstanding usability for tourists, and visitor security, with specific references to visitor utilizing incapacities. The GA was utilized for designing a combined security system, especially for the field of components such as CCTV cameras, Wi-Fi Access Points, and installation poles for ensuring final cost reduction and a higher level of consistency and flexibility of the scheme for themselves for deliberation: the usual vincula and restriction of archaeological regions. Babou et al. [25] proposed a solution for resolving the problem of delays caused by resource constraints on HEC servers. Increased traffic causes server waits, which increases the processing time (delay) for the request. They suggested a unique HEC Clustering Balance approach using leverage, clustering, and LB methods. It enables hierarchical request allocation on the HEC cluster and other framework components to minimize congestion on the HEC server and reduce latency. Table 1 elaborates on the state of artworks related to existing works. Based on their findings, Singh et al. [26] compared and contrasted various load-balancing strategies, classification schemes, and algorithms. According to the authors’ research, round robin is the easiest load-balancing technique in a fog computing system. IP Hash was employed as the load-balancing method in the fog computing infrastructure. Another technique for finding malicious nodes was presented by Sangaiah et al. [27]: “Clustering Multi-Layer Security Protocol (CL-MLSP)” combined with “Ad-hoc On-Demand Distance Vector” (AODV). In terms of mobility, dispersion, and energy, the clustering method calculated the shortest distance between each node. The CL effectiveness MLSPs were measured across a range of metrics, including network lifetime, packet loss, latency, and security, using NS2.

The system attempted to prevent a scenario where a node and its neighbors used the same routing path to their respective destinations. The load balancing impact was boosted because the next hop was selected using satellite ephemeris prediction and traffic allocation. A load-balancing routing technique built on extended link states was proposed by C. Dong et al. [28]. With a system of active state discovery and automated congestion state release, they ensure that all satellite nodes are aware of the current condition of the link. All satellite nodes must adjust their route tables in real-time to ensure that traffic is evenly distributed over available connections.

**Table 1:** Features and survey of existing methodologies.

Reference	Method/Techniques	Features	Limitations
Cao et al. [17]	EdgeOSH	It also addressed non-functional issues, such as a lack of open testbed availability, latency, system cost, and user experience, and functional ones, such as self-management, programming interface, security and privacy, nomenclature, and data management.	The study did not evaluate the performance of the proposed method.
Naranjo et al. [18]	FOCAN	Using various abilities, the FOCAN lowers delay and improves energy provision and service efficacy between objects. With FOCAN devices, There are three categories of communications: primary, secondary, inter-primary, and transmission for controlling applications that fulfil the standard QoS for IoE.	It proposes a fog-based smart city network architecture without an algorithm.
Singh et al. [19]	Blockchain and Fog-based Architecture Network (BFAN)	The framework aims to reduce time while providing increased security via Blockchain.	It does not provide any specific implementation details or algorithm.
Divyabharathi et al. [20]	Map-Reduce	It offers the PUF chain a unique blockchain concept that integrates hardware security primitives known as PUFs for overcoming latency, scalability, and energy needs.	It very briefly discusses the framework only without the implementation aspect and comparison.
Miao et al. [21]	Fair and dynamic data sharing framework (FairDynDSF)	This method could validate search results, run multi-keyword searches, make dynamic upgrades, and accomplish fair arbitration.	It mainly deals with security aspects and does not detail resource optimization.
Xiao et al. [22]	Nash bargaining solution (NBS)	The method varies from work theft scheduling because it is based on the NBS for cooperative games among FCNs. FCNs collaborate to increase the efficiency of all FCNs to reach Pareto optimality.	It only focuses on a work-stealing algorithm (GWS) and a classical work-stealing algorithm (CWS) for resource optimization.
Chithambaramani et al. [23]	Hybrid squirrel search genetic algorithm (HSSGA) and improved adaptive neuro-fuzzy inference system (I-ANFIS)	The computational offloading issues of the existence and synergy between FC and CC in IoE were investigated by jointly enhancing the offloading decision, the distribution of computational assets, and transfer power.	It mainly deals with cloud computing environments and does not compare results with other algorithms.
Babou et al. [25]	Genetic Algorithms (GAs)	The increase in traffic causes a large wait on these servers, which increases the processing time (delay) for the request. They proposed a novel strategy dubbed HEC Clustering Balance, which combines leverage, clustering, and LB approaches. It allocates hierarchical requests on the HEC cluster and other framework components to reduce HEC server congestion and latency.	It does not compare results with available literature.
Jeyaraj et al. [29]	Systematic review	This article systematically reviews resource management tasks in the cloud for IoT applications.	It is a systematic review, but no detailed information is given for any algorithm.
Mehran, Izadi, and Ghobaei-Arani [30]	Micro-genetic algorithm	This article provides a Micro-genetic algorithm-based mechanism to decide the locations of various virtual machines (VMs) of the cloud data center to minimize power consumption.	It deals with cloud data centres and does not deal with IoT or IoE nodes directly.
Tanzila, et al. [31]	Particle swarm optimization	It proposes a particle swarm optimization-based load-balancing mechanism in the IoE cloud platform to reduce latency and transmission overhead.	It is well written but does not consider ML/DL for resource optimization.
Ghobaei-Arani and Shahidineja [32]	Whale optimization algorithm (WOA)	It proposes a WoA-based QoS monitoring platform for IoT services and provides a mechanism for service placement in the fog-cloud environment.	It deals with only metaheuristic-based mechanisms
Farahbakhs, Shahidinejad and Ghobaei-Arani [33]	Bayesian learning automata (BLA)	This article proposes a context-aware offloading approach using Bayesian learning automata in mobile edge computing for Internet of Things applications, aiming to optimize performance metrics and improve offloading efficiency in multiuser scenarios.	It does not compare results with available literature.
Quy, Hau, Anh, and Ngoc [34]	Review article on Fog-IoHT	This article discusses the limitations of cloud-based healthcare applications. It proposes a fog-computing-based architectural framework for Internet of Health Things (IoHT) applications, highlighting its potential and addressing challenges in integrating fog computing into healthcare IoT.	It is a review article on Fog-IoHT; no detailed information is given for any algorithm and resource optimization.
Khanh et al. [35]	Information map	This study proposes an efficient edge computing management mechanism using an information map to reduce service response times and improve energy consumption in IoT applications for smart cities, aiming for its future application in sustainable smart cities.	It is the latest article but mainly deals with smart city concepts.

## 3. Background Study

The IoE is spurring the development of various systems and applications that are capable of collecting and analyzing vast amounts of data. Sensors and devices are typically used to gather this data, which consume considerable amounts of power, necessitating the development of low-power data retrieval systems [36]. Several studies have proposed various architectures and techniques to build such systems. One study by [37] proposed a low-power data retrieval system for IoT that combines wireless and power line communication (PLC) to acquire and transmit remote data with minimal power consumption. The authors demonstrated the effectiveness of their system in temperature and humidity monitoring [38] presented a wireless data acquisition system for IoT that utilized a Bluetooth Low Energy (BLE) communication protocol and evaluated its performance in terms of power consumption, transmission range, and data rate in a smart home application. Paper [39] designed a low-power wireless sensor network (WSN) for smart agriculture based on the ZigBee protocol, powered by solar panels, and tested it in real-world applications of soil moisture monitoring. Article [40] provided a review of IoT-facilitating technologies, including various wireless communication tools and their suitability for IoT applications. Article [41] proposed a low-power WSN for use in agriculture based on the ZigBee protocol for real-time soil moisture, temperature, and humidity monitoring. Finally, [42] proposed a low-power WSN for smart greenhouse monitoring using the ZigBee protocol, designed for real-time monitoring of various environmental parameters, including temperature, humidity, light, and carbon dioxide, and tested it in a real-world application.

A tabular comparison of these six studies is provided in Table 2 in terms of their communication protocols, power supply, real-world applications, and reported results, highlighting the varied approaches and outcomes of these studies.

Based on this comparison, it can be seen that each study has its own unique features and advantages. For example, study [43] uses a low-cost and open-source platform, while study [38] offers powerful processing capabilities. Study [39] prioritizes secure communication using TLS/DTLS, and study [40] has the added benefit of BLE connectivity and a dual-core processor. Study [41] focuses on low-power consumption and onboard Wi-Fi, and study [42] boasts a very low power consumption of only 0.42 milli watts.

Overall, the choice of IoT platform and communication protocol will depend on the specific needs and requirements of the data retrieval system. Factors such as power consumption, cost, processing capabilities, and security should all be considered when selecting the most suitable platform and protocol for a given application. The studies mentioned above propose different techniques and architectures for building low-power data retrieval systems for IoT applications. These systems require efficient algorithms to manage the collected data and optimize the system’s performance. It is where the CHROA optimization algorithm is applied to improve the data retrieval scheme.

The Chaotic Horse Ride Optimization Algorithm (CHROA) is an advanced optimization algorithm that combines the principles of chaos theory with the Horse Ride Optimization Algorithm (HROA). CHROA aims to enhance the optimization capabilities of HROA by introducing chaotic behavior. It utilizes chaotic maps to introduce randomness and unpredictability into the search process, improving search space exploration. CHROA generates an initial population of potential solutions and iteratively updates its positions based on fitness evaluations. The chaotic maps influence the position updates, diversifying the search and balancing exploration and exploitation. Experimental results show that CHROA outperforms other algorithms in terms of solution accuracy and convergence speed. In cloud-enabled IoT environments, CHROA can be used for efficient workload distribution, energy resource optimization, and improved system performance. It offers an intelligent, energy-aware approach to load balancing in IoT/IoE applications.

Applying this algorithm makes optimizing the data retrieval system’s performance possible by reducing power consumption, enhancing data transmission rates, and improving data accuracy. For example, CHROA can be used to optimize the design of WSN used in smart agriculture, as demonstrated in the studies by [39,40]. The algorithm can be used to optimize the placement of sensors, reduce energy consumption, and improve the accuracy of data collection.

Similarly, the CHROA algorithm can be applied to optimize the performance of low-power data retrieval systems, such as those proposed by [38,39,43], by optimizing communication protocols, data transmission rates, and power consumption. The CHROA optimization algorithm can be applied to optimize the performance of low-power data retrieval systems in IoT applications. The algorithm can be used to optimize the design and operation of WSN, reduce power consumption, and enhance data transmission rates and accuracy.

Limited literature is available on applying the CHROA optimization algorithm for optimizing the performance of low-power data retrieval systems in IoT applications. However, some recent studies have shown promising results in this area. Paper [44] proposed a novel method for optimizing the energy consumption of low-power data retrieval systems in IoT applications using the CHROA optimization algorithm. The authors evaluated their approach using a real-world application of temperature and humidity monitoring and reported significant improvements in the energy efficiency.

Similarly, Ref. [45] presented a study on optimizing the performance of a WSN using the CHROA optimization algorithm. The authors focused on minimizing the network’s energy consumption while maintaining a certain level of coverage and connectivity. The results showed that their approach significantly improved the energy efficiency of the network.

In another study, Ref. [46] applied the CHROA optimization algorithm to optimize the low-power WSN routing. The authors focused on minimizing the network’s energy consumption while maintaining reliable communication between the nodes. The results presented in Table 3 showed that their approach outperformed traditional routing protocols in terms of energy efficiency.

## 4. Proposed Model

**Figure 2:**
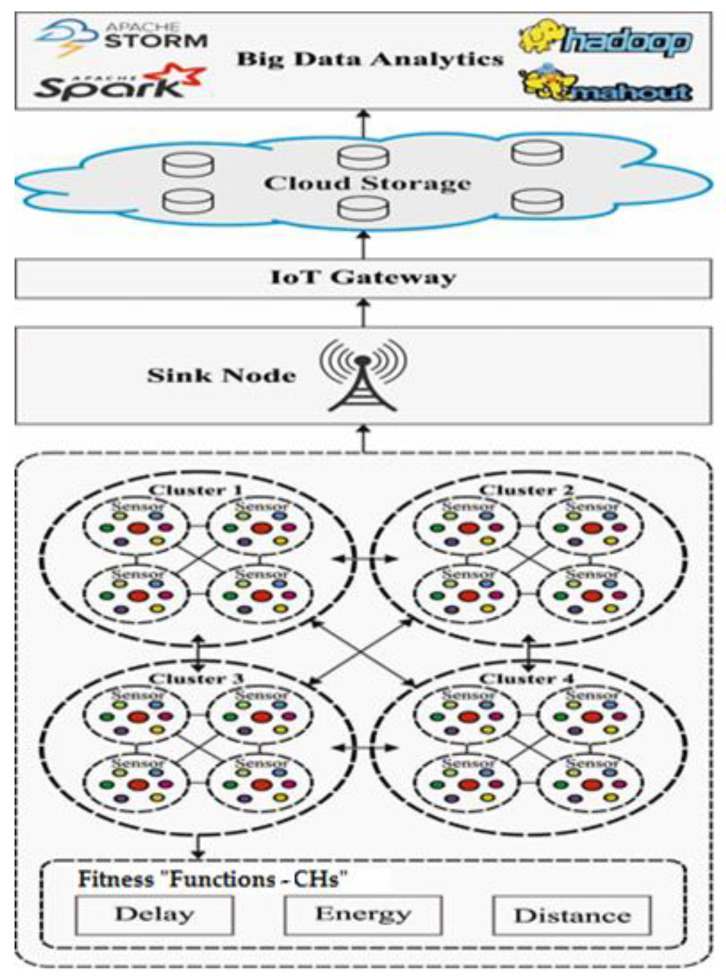
Working process of CHORA model.

Figure 2 depicts the overall system design of the model, where the IoE system comprises a set of clusters generated by the CHROA technique. Every cluster includes a cluster head (CH) and related cluster members (CMs).

Moreover, the CMs send the data to the CHs, which are forwarded to the IoE BS. The CHROA technique involves three major steps in offering real-time services as listed in the following:

- CHROA-based clustering of the IoT networks,
- Cloud Data Storage and Processing,
- Client Services.

The following sections provide a detailed explanation of how these modules work.

### 4.1. Algorithmic Design of CHROA Technique

The CHROA technique is mainly based on HROA and chaos theory concepts. Chaos is in an unbalanced state by paying attention to early conditions. It avoids optimal local difficulties and improves the quality of solutions in various optimization procedures.

**Figure 3:**
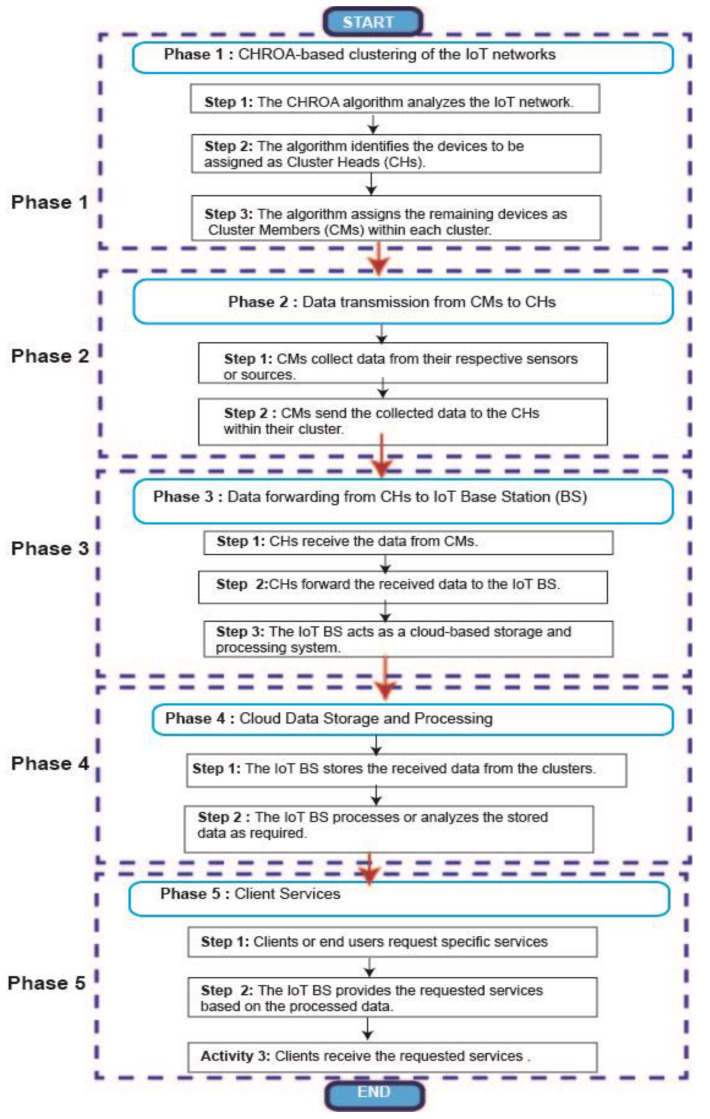
Flow diagram of CHROA algorithm.

Unlike a pendulum, a chaotic system cannot settle into a regular rhythm due to its nonlinear dynamics. Using hierarchical horse herds promotes the CHROA. A horse is an example of a herd animal. Because many animals congregate in big groups, which reduces conflict and improves social cohesion, a stable hierarchical structure or “pecking order” is preferred. This type of linear system is used on occasion. In nonlinear hierarchies, Horse A may dominate Horse B, Horse B may dominate Horse C, and so on. Dominance might be centered on a range of elements, such as a person’s desire for certain possessions at a given moment. It might change during a herd’s or individual animal’s lifecycle. Humans force horses to coexist in a confined location with few resources. Figure 3 shows the flow diagram of the CHROA algorithm.

CHROA is referred to as “dominant horses” with dysfunctional social capabilities. When grain is scarce, higher-status horses may ban lower-status horses from blocking animals of lower social rank from eating at all. Animals with a lower social rank who eat later risk not receiving enough food.

Access to resources is prioritized in a horse herd headed by a dominant stallion and mare, depending on the horses’ hierarchical positions within the herd [7]. Initially, the hierarchy of the horses in a herd is defined by their fitness levels. Consider a herd of k horses, where P represents the function.

(1) $$Herd=\left\{H_{1},\ldots ,H_{k}\right\}$$

(2) P=Herd→1,…,

When fitness $\left(H_{x}\right)$ < fitness $\left(H_{y}\right)$, whereas x≠y and x, yϵ{1…K}, then

(3) $$P\left(H_{x}\right)>P\left(H_{y}\right)$$

When fitness $\left(H_{x}\right)$ = fitness $\left(H_{y}\right)$, whereas x≠y and x, yϵ{1…K}, then

(4) $$\left[P\left(H_{x}\right)-P\left(H_{y}\right)\right]\left(x-y\right)>0\;$$

The rank of every horse $H_{x}$ is defined as follows:

(5) $$H_{x}-Rank\;of\;each\;horse=\frac{P\left(H_{x}\right)}{K}$$

Every herd has a center that is equivalent to the weighted average of the positions of the horse from the herd, thus, the weights are denoted as a rank of the horse. The center of the herd is estimated by:

(6) $$Herd_{Center}=\frac{\sum_{x=1}^{k}Z_{x}H_{x.rank}}{\sum_{x=1}^{k}H_{x.rank}}$$

Every herd has a center that corresponds to the weighted average of the positions of its horses; thus, the weights represent the horse’s position in the herd. The approximate position of the herd’s center is listed below:

(7) $$Dim\left(Stallion,herd\right)=\sqrt{\overset{Dim}{\underset{y=1}{\sum }}{\left(Staalion_{y}-Herd_{Center}\right)}^{2}}$$

Whereas Dim denotes the number of dimensions of the search space. When the horses belong to the group herd of the horse then it upgrades its velocity by:

(8) $$Vel_{x,y}^{T+1}=Vel_{x,y}^{T}+H_{x.rank}\times \left(Herd_{center.y}^{T}-Z_{x,y}^{t}\right)$$

(9) $$Vel_{x,y}^{T+1}=Vel_{x,y}^{T}+Rand\times \left(Herd_{center.y}^{T}-Z_{x,y}^{t}\right)$$

where Rand denotes an arbitrary number from zero to one. T indicates the present iteration, and T+1 represents a novel iteration. The memory of a horse (*Mem*) is a matrix that has a number of rows equivalent to the value of the Horse Memory Pool (*HMP*) and *D* columns.

(10) $$Mem_{x}^{T+1}=\left[\begin{array}{ccc} Mem_{1,x,1}^{T+1} & \cdots & Mem_{1,x,D}^{T+1}\\ \vdots & \ddots & \vdots \\ Mem_{HMP,x,1}^{T+1} & \cdots & Mem_{HMP,x,D}^{T+1} \end{array}\right]$$

The formula for updating the cell of the memory matrix is,

(11) $$Mem_{K,x,y}^{T+1}=Z_{x,y}^{T+1}\times N\left(0,SD\right)$$

where N represents a normal distribution, with 0 acting as the mean and SD serving as the standard deviation.

Algorithm 1 provide a Pseudo Code for the CHROA Technique for a reference that can be implemented in programming language.
**Algorithm 1:** Pseudo Code of CHROA TechniqueBeginning of Algorithm 1    Initiate the horse population in the search space     Calculate fitness of the horse and upgrade the optimal one   While iteration < T      Define the center of every herd from T    For n = 1 to number of horse    Find hbest    Find cbest    For T = 1 to horse velocity      Estimate the rank of every horse from top to bottom      Upgrade the horse velocity and location      Calculate the fitnessnext l        next n   If fitness condition is fulfilled      End     T = T+1, Return the Global optimum solution  End While     End of Algorithm.

The chaos idea is incorporated to enhance the global optimization capability of the HROA method. Chaos is an imbalanced state that is very sensitive to the initial circumstances. It is used in a variety of optimization approaches to avoid optimal local issues and improve solution quality [47]. As the metaheuristic technique is based on exploration and exploitation phases, the chaos idea is presented for maintaining an efficient tradeoff between exploitation and exploration, thus attaining an optimal solution efficiently. In HROA, variable $H_{x}$ has a significant influence on the convergence rate of the Artificial Fish Optimization (AFO) method. The HROA method’s efficiency depends on its variables. It might be observed by a large momentum to begin utilizing the potential search space, and it cannot be revealed effectively. The chaos is utilized for attaining improved search features for the exploitation and exploration area, thus improving the result to identify optimal global results. The chaotic map is applied to define the position $x_{i}^{k}$, whereas the parameter θ substituted with values attained using chaotic map is given by,

(12) $$x_{i}^{k+1}=x_{i}^{k}+C_{nap}\times \left(x_{BH}-x_{i}^{k}\right),i=1,2,\ldots ,N_{\nabla }$$

where, $x_{BH}$ denotes the location of BH from space, $C_{map}$ indicates the chaotic map, and $N_{\nabla }$ denotes the total number of individuals in the optimization algorithm. In short, ten chaotic maps are used to manipulate the value of random variables from the HROA method.

### 4.2. Design of Objective Function

The objective function of the presented study is determined in Equations (13)–(15). The term (β,γ) indicates the constant with a fixed value of (0.9, 0.3).

(13) $$OM_{1}=O_{M}^{ene}\frac{1}{O_{M}^{load}}+O_{M}^{ene}\frac{1}{O_{M}^{temp}}$$

(14) $$OM_{2}=\beta \frac{1}{O_{M}^{dist}}+\left(1-\beta \right)OM_{1}$$

(15) $$OM_{3}=\gamma OM_{2}+\left(1-\gamma \right)\frac{1}{O_{M}^{del}}$$

Equation (16) provides the sensor node’s distance to the BS. The distances between the CHs and BS is defined by Equation (17). Likewise, $O_{M}^{dist}\left(n\right)$ examines the distance between 2 nodes that should lie in the range [0, 1].

$O_{M}^{dist}\left(m\right)$ denotes the distance between the sensor node and CH represented by [24]:

(16) $$O_{M}^{dist}=\frac{O_{M}^{dist}\left(m\right)}{O_{M}^{dist}\left(n\right)}$$

(17) $$O_{M}^{dist}\left(m\right)=\overset{N}{\underset{p=l}{\sum }}\overset{NCH}{\underset{q=1}{\sum }}\|N_{p}^{normal}-CH^{q}\|+\|CH^{q}-B^{I}\|$$

(18) $$O_{M}^{dist}\left(n\right)=\overset{N}{\underset{p=1}{\sum }}\overset{NCH}{\underset{q=1}{\sum }}\|N_{p}^{norm\alpha l}-N_{q}^{normal}\|$$

Next, Equation (19) calculates the energy utilization by IoT devices. For improving the network efficiency, the energy must be maximal as given in Equation (22):

ENE $\left(N_{p}^{normal}\right)$ specifies an energy of $p^{th}$ normal node.

ENE $\left(N_{q}^{normal}\right)$ denotes an energy of $q^{th}$ normal node.

(19) $$O_{M}^{ene}=\frac{O_{M}^{ene}\left(m\right)}{O_{M}^{ene}\left(n\right)}$$

(20) $$O_{M}^{ene}\left(m\right)=\overset{CH}{\underset{q=1}{\sum }}nENE\left(q\right)$$

(21) $$nENE\left(q\right)=\overset{M^{M}}{\underset{p=1,p\varepsilon q}{\sum }}\left(1-ENE\left(N^{normal}\right)\times ENE\left(CH\right)\right);1\leq q<CH$$

(22) $$\begin{array}{c} O_{M}^{ene}\left(n\right)=CH\times Max_{p=1}^{CH}\left(ENE\left(N_{p}^{normal}\right)\right)\\ Max_{q=1}^{CH}\left(ENE\left(N_{q}^{norm\alpha l}\right)\right)\; \end{array}$$

Finally, Equation (23) is utilized for measuring the delay endured by IoT devices while transferring data to the BS. The number of sensor nodes in every cluster should be smaller to avoid delay. $Max\left(CH^{q}\right)$ represents the number of CHs in the network.

$N^{N}$ indicates the cumulative IoT sensors.

(23) $$M_{del}=\frac{Max\left(CH^{q}\right)}{N^{N}}$$

### 4.3. Cloud Data Storage and Processing

**Figure 4:**
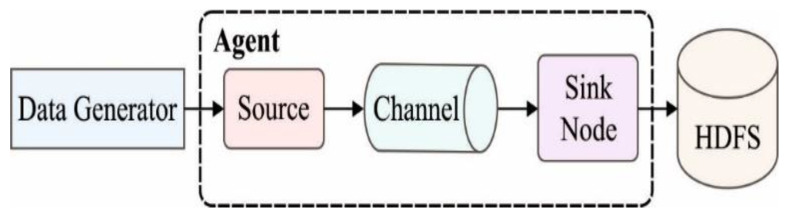
Apache Flume.

Cloud storage plays a crucial role in the proposed load-balancing model for the cloud-enabled Internet of Everything (IoE) in the healthcare domain. Cloud storage is a specific type of cloud computing architecture where a cloud computing provider stores data over the Internet. This approach eliminates the need for organizations to build and maintain their own data centers, reducing costs and providing storage space on demand [36]. By utilizing cloud data management, the proposed model ensures that data are securely stored on servers located in separate locations and managed by specialized cloud data hosting providers. This enables benefits such as automated backups, expert assistance, and remote access to data [48]. In the proposed model, data are processed and sent to the Gateway (GT) before being stored in the cloud. Apache Flume, a Big Data tool, consumes and ingests the data. The data are then stored in the Hadoop Distributed File System (HDFS) for efficient and scalable storage [49]. This integrated approach leverages the capabilities of cloud storage, Apache Flume, HDFS, and big data analytics efficiently handle and analyze the massive volume of real-time streaming data generated by IoT devices in the healthcare domain [50]. The combination of cloud computing and the Internet of Everything (IoE) enables the delivery of real-time services. In the context of IoT devices, big data analytics tools are utilized to process and organize unstructured data from various sources, such as sensors and social media, into consumable datasets. These data can be used to derive insights and improve operational efficiency in diverse areas, ranging from traffic patterns to home efficiency [48]. The proposed load balancing model incorporates Apache Flume, a scalable technique for large-scale real-time data streaming in the context of Big Data. Apache Flume ensures a balanced flow of data by dynamically adjusting the write and read rates to accommodate varying data generation and consumption speeds [51]. The essential architecture of Apache Flume is depicted in Figure 4.

The Flume framework consists of agents, events, and clients. Flume events are the fundamental units of data transmitted through the Flume pipeline and stored in HDFS. Flume agents collect data from clients and transmit them from sources to sinks, using sources, channels, and sinks as essential components. Flume clients generate data events and send them to agents for storage in the HDFS platform.

Furthermore, the proposed model incorporates Apache Spark, a cluster-based solution, for real-time processing. Apache Spark has gained significant popularity for its ability to handle Big Data challenges with fast computation. The model’s Algorithm 2 combines the chaotic map equation and the Horse Ride Optimization Algorithm (HROA) technique to generate new individuals and explore the search space efficiently. Overall, the proposed load balancing model utilizes the capabilities of cloud storage, Apache Flume, HDFS, and Apache Spark to handle large-scale real-time data streaming, optimize resource allocation, and provide efficient processing in cloud-enabled Internet of Everything (IoE) environments. Algorithm 2 provide a Pseudo Code for CHROA technique incorporating cloud data storage and processing.
**Algorithm 2:** CHROA technique incorporating cloud data storage and processing**Input: **  - n: number of entities  - MaxIter: max no. of iterations  - p: probability of chaos  - alpha: chaos parameter  - objective function f(x) that involves processing large amounts of data stored in the cloud**Output:**  - x_best: the best solution found  - f_best: the corresponding fitness value**Initialization:**  - Initialize the population x_i, i = 1, …, n with random positions**Evaluation:**- Evaluate the fitness of each individual using the objective function f(x), which may involve processing large amounts of data stored in the cloud**Selection:**- Select the best individuals based on their fitness values**Iteration:**  - For iter = 1 to MaxIter do:    - Generate new individuals using the chaotic map equation and the HROA technique:      - Select two entities x_a and x_b arbitrarily from the selected best individuals      - Generate a fresh entities x_new using the chaotic map equation:        x_new = x_a + alpha × (x_b − x_a) × r        where r is a random no. between −1 and 1      - Apply the HROA technique to the new individual:        x_new = x_new + p × D × (x_best − x_new) × r        where D is the variance between the max and min positions in the population      - Evaluate the fitness of the new individual using the objective function f(x), which may involve processing large amounts of data stored in the cloud      - Select the best entity from the collective populace of old and new entities- End for**Output:**  - x_best: the best solution found  - f_best: the corresponding fitness value

### 4.4. Client Service

The proposed framework enables machine-to-machine interaction. In the healthcare domain, various end users, such as patients, doctors, nursing staff, etc., can request and access gathered and monitored data stored in the cloud. Service requests are handled seamlessly regardless of origin, as the collected data are stored in the cloud. An integral component of the model is the Event Aware Node (EAWN), which is responsible for sending requests to the Database Server (DBS). The DBS, in turn, forwards these requests to the IoE network [52]. This intelligent transmission facilitated by the EAWN significantly reduces the turnaround time and improves the throughput of the system [53]. With the EAWN’s awareness of events and its efficient forwarding of requests to the DBS, the load-balancing model achieves a notable decrease in both the turnaround time and throughput, enhancing the system’s overall performance.

## 5. Performance Analysis

**Figure 5:**
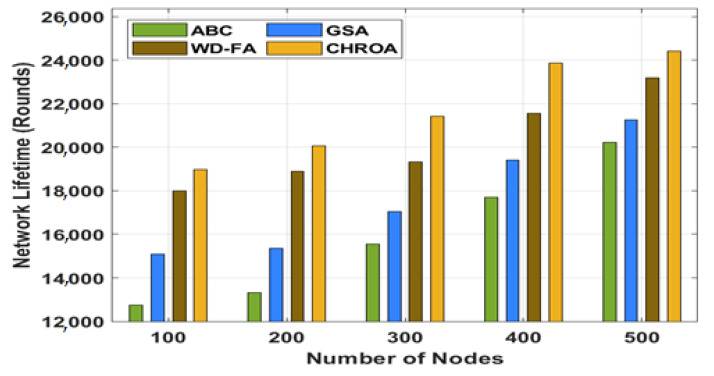
Network lifetime analysis of CHROA model with existing techniques.

The experimental results of the proposed CHROA model are validated using the MATLAB tool. A network with a grid size of 5003 × 500 m is considered with a total of 500 nodes. Moreover, a detailed comparative analysis is made with the existing ABC, GSA, and WD-FA techniques (Table 4 and Figure 5). This section compares the CHROA technique to other current practices with varied numbers of nodes, and the NLT value should be high for better results. The figure portrayed that the ABC algorithm shows a poor performance with the minimum NLT, whereas the GSA and WD-FA techniques have demonstrated moderate outcomes. The proposed CHROA strategy, however, has achieved the highest levels of NLT of any of the approaches considered. With 100 nodes, for instance, the NLT for the CHROA method is 18,972 rounds, while the NLTs for the ABC method (12,739 rounds), the GSA method (15,101 rounds), and the WD-FA method (17,995 rounds) are all lower. With 500 nodes, the NLT for the CHROA technique has gone up to 24,404 rounds, while the NLT for the ABC, GSA, and WD-FA techniques has gone down to 20,225, 21,265, and 23,193 rounds, respectively.

Figure 5 inspects CHROA’s performance compared to the existing methods in terms of the TEC, and the value of the TEC needs to be low for better performance. For example, with 100 nodes, the CHROA approach provides a minimum TEC of 6.84 J, while the ABC, GSA, and WD-FA procedures produce maximum TECs of 10.70 J, 10.05 J, and 8.55 J, respectively. Finally, with 500 nodes, the CHROA approach has a lower TEC of 13.84 J, but the ABC, GSA, and WD-FA techniques exhibit higher TECs of 21.28 J, 19.56 J, and 15.82 J, respectively.

**Figure 6:**
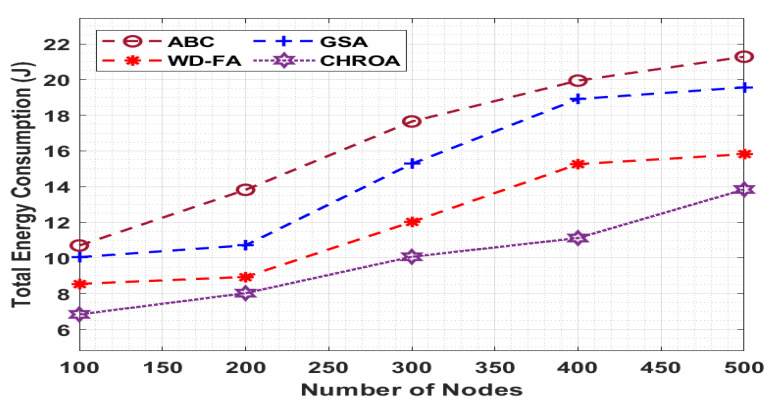
Total energy consumption analysis of CHROA model.

In Figure 6, the throughput of the CHROA technique is compared to different models with variable nodes, and the value of throughput must be larger to improve outcomes. The graph demonstrated that the ABC method performed poorly with low throughput, but the GSA and WD-FA methods surpassed intermediate results. However, the provided CHROA algorithm outperformed the other strategies in terms of throughput.

**Figure 7:**
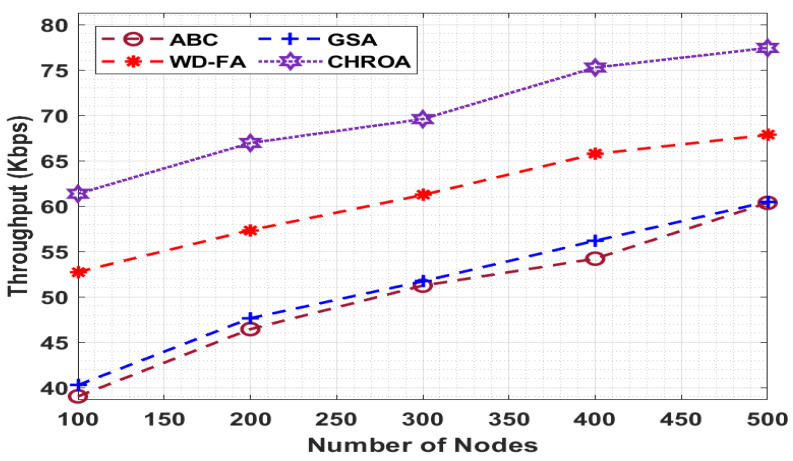
Throughput analysis of CHROA model with existing techniques.

Figure 7 shows that with 100 nodes, the CHROA technique achieved a higher throughput of 61.36 Kbps, whereas the ABC, GSA, and WD-FA methods achieved lower throughputs of 39.04 Kbps, 40.28 Kbps, and 52.75 Kbps, respectively. Similarly, with 500 nodes, the CHROA technique obtained a higher throughput of 77.43 Kbps, but the ABC, GSA, and WD-FA approaches achieved lower throughputs of 60.36 Kbps, 60.48 Kbps, and 67.85 Kbps, respectively. Table 5 briefly compares the CHROA technique with other known algorithms at various node counts.

**Figure 8:**
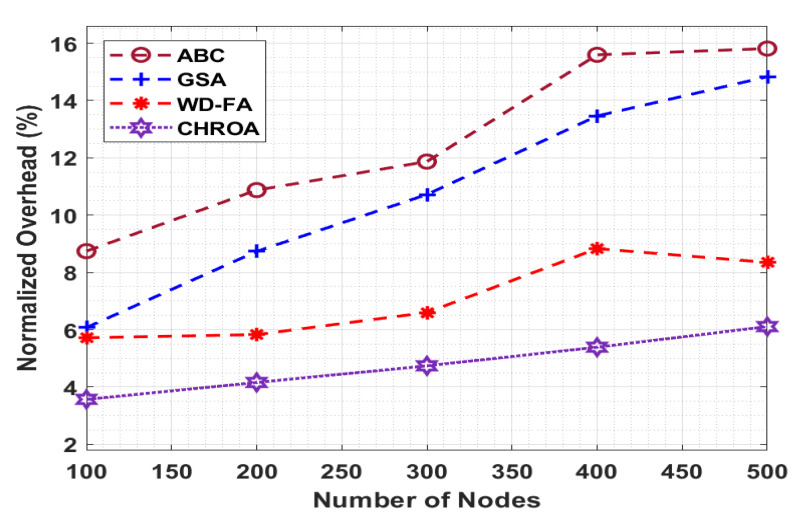
Normalized overhead analysis of CHROA model with existing techniques.

Figure 8 compares the performance of the provided CHROA model to previous methods in terms of the NO, and the value of the NO must be lower for the best performance. The figure shows that the CHROA technique has achieved maximum performance with reduced NO levels. For example, with 100 nodes, the CHROA algorithm provides a minimum NO of 3.570%, whereas the ABC, GSA, and WD-FA techniques provide a greater NO of 8.742%, 6.075%, and 5.720%, respectively. Finally, with 500 nodes, the CHROA technique achieves a minimum NO of 6.110%, but the ABC, GSA, and WD-FA algorithms achieve better NOs of 15.813%, 14.83%, and 8.350%, respectively.

**Figure 9:**
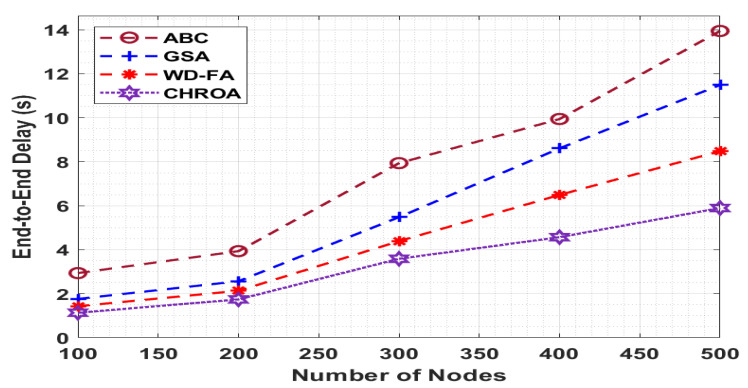
Comparison of ETE delay of CHROA with current techniques.

Figure 9 determines the performance of the projected CHROA manner with existing approaches in terms of the ETE delay, and the minimum value of the ETE delay gives optimal performance. The figure shows that the CHROA method has gained higher performance with the minimum values of ETE delay. For the sample with 100 nodes, a lower ETE delay of 1.136 s exists by the CHROA manner, whereas a maximal ETE delay of 2.938 s, 1.764 s, and 1.431 s have been offered by the ABC, GSA, and WD-FA methods, respectively. At last, with 500 nodes, a minimum ETE delay of 5.896 s is outperformed by the CHROA approach, whereas a maximum ETE delay of 13.945 s, 11.490 s, and 8.481 s have been reached by the ABC, GSA, and WD-FA methodologies, respectively.

**Figure 10:**
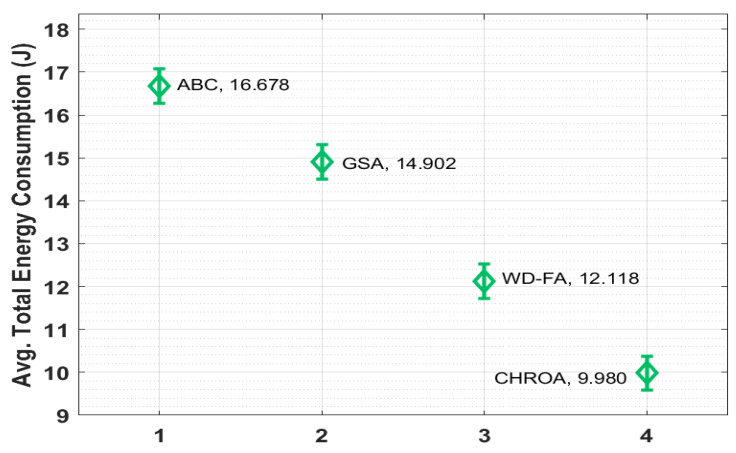
Comparison of avg. TEC of CHROA model with current techniques.

Figure 10 shows the average TEC (Total Energy Consumption) analysis of the CHROA model. According to the data, ABC has a poor average TEC of 16.678 J, whereas the GSA technique has a slightly increased average TEC of 14.902 J. In addition, the WD-FS technique has accomplished a moderate TEC of 12.118 J. However, the suggested CHROA approach resulted in effective network performance with an average TEC of 9.980 J, which is the lowest achievable value.

**Figure 11:**
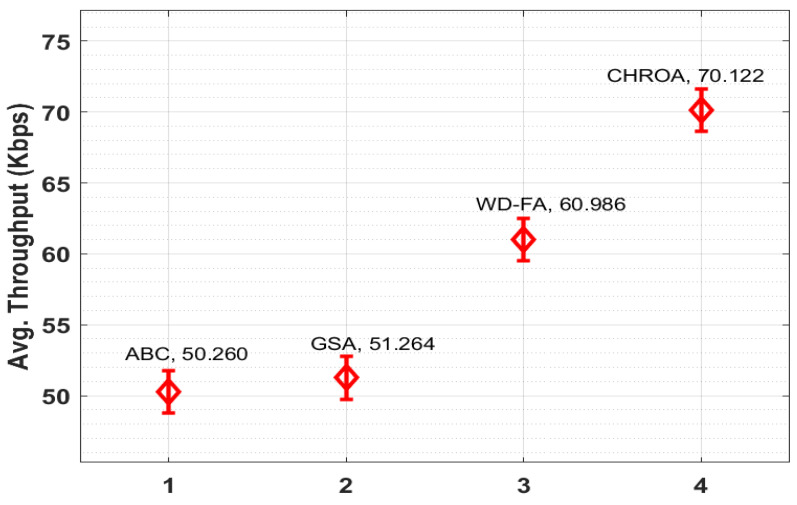
Comparison of avg. throughput of CHROA model with current techniques.

Figure 11 compares the CHROA’s average throughput to several techniques. ABC and GSA have minimal average throughputs of 50.260 Kbps and 51.264 Kbps, respectively. Moreover, the WD-FA technique depicted a moderate average throughput of 60.986 Kbps. With an average throughput of 70,122 Kbps, the proposed CHROA method exceeds the competition. The tables and graphs below demonstrate CHROA’s effectiveness as a cloud-based IoE load balancer [26].

## 6. Comparison of CHROA with Existing Techniques

Table 6 presents a comparison of the performance metrics of four optimization algorithms, including the CHROA, ABC, GSA, and WD-FA.

In terms of the average TEC (mJ/bit) metric, which measures the total energy consumed by the network to transmit one bit of data, CHROA outperformed the other three algorithms, with the lowest average TEC of 0.1736 mJ/bit. The average throughput (Kbps) metric, which measures the amount of data transmitted per unit of time, was also highest for CHROA, with an average throughput of 70.122 Kbps. ABC, GSA, and WD-FA had average throughputs of 58.247 Kbps, 59.957 Kbps, and 60.819 Kbps, respectively. The ETE delay (s) metric, which measures the end-to-end delay in data transmission, was also lowest for CHROA, with a delay of 0.0643 s. ABC, GSA, and WD-FA had ETE delays of 0.0781 s, 0.0716 s, and 0.0752 s, respectively. The normalized overhead metric, which measures the number of control messages required to maintain the network, was lowest for CHROA, with a normalized overhead of 0.4069. ABC, GSA, and WD-FA had normalized overheads of 0.4808, 0.4437, and 0.4784, respectively.

In terms of the network lifetime (s), which measures the duration for which the network can operate efficiently, CHROA outperformed the other three algorithms, with a network lifetime of 510.256 s. ABC, GSA, and WD-FA had network lifetimes of 445.561 s, 481.725 s, and 467.865 s, respectively. The total energy consumption (Joule) metric, which measures the total energy consumed by the network, was lowest for CHROA, with a total energy consumption of 5.1289 Joule. ABC, GSA, and WD-FA had total energy consumptions of 6.7855 Joule, 6.2234 Joule, and 6.7464 Joule, respectively.

Finally, in terms of the throughput (Kbps) metric, which measures the amount of data transmitted per unit of time, CHROA again outperformed the other three algorithms, with a throughput of 69.485 Kbps. ABC, GSA, and WD-FA had throughputs of 55.621 Kbps, 58.072 Kbps, and 58.961 Kbps, respectively. Overall, CHROA outperformed ABC, GSA, and WD-FA in most of the performance metrics evaluated, indicating that it is an effective optimization algorithm for energy saving and load balancing in cloud-based IoE environments.

To gain further insight into the data, Analysis of Variance (ANOVA) and the Wilcoxon signed-rank test were conducted on the results available in Table 6 and the analyses were presented in Table 7 and Table 8, respectively.

We have considered that:

- Null Hypothesis (H0): There is no significant difference in the medians of the algorithms.
- Alternative Hypothesis (H1): There is a significant difference in the medians of the algorithms.

The *p*-value for the Average Throughput metric was less than 0.05, which means we could reject the null hypothesis. Therefore, we can conclude that there is a significant difference between the means of the three groups for the Average Throughput (Kbps) metric.

The *p*-value for the Average Throughput metric was less than 0.05, which means we could reject the null hypothesis. Therefore, we can conclude that there is a significant difference between the means of the two groups for the Average Throughput (Kbps) metric.

Therefore, further based on the Analysis of Variance (ANOVA) and Wilcoxon signed-rank test, it is clear that there is a significant variation in the Average Throughput; hence, CHROA is outperforming the other algorithms significantly.

## 7. Future Research Work

While this research presents a promising approach to optimizing the CHROA algorithm for cloud-based IoE environments, there is still room for further investigation and improvement. Future research could focus on developing AI-based performance enhancement strategies for the system to optimize energy savings and load balancing further. Additionally, integrating UAVs with the IoE to overcome network coverage and resource constraints should be explored further, along with developing routing protocols based on contextual information to enhance the network performance. Moreover, load-balancing strategies supported by clustering could be further studied to more efficiently manage IoT activities while considering energy and performance requirements. Further traffic shaping and traffic policing techniques can be utilized to improve QoS and High-Available Proxy (HA proxy) for improving the system reliability and load balancing. The proposed dynamic clustering approach based on vehicle position, velocity, and heading is effective even for UAVs, but more research could be conducted to optimize its performance. Overall, further research could lead to developing more effective and efficient energy-saving and load-balancing approaches in cloud-based IoE environments.

## 8. Conclusions

The research suggests a novel energy-saving and load-balancing approach in cloud-based IoE environments using the CHROA. The CHROA method employs a fitness function with three parameters to optimize the selection of CHs and cluster construction, while Apache Flume is used as a Big Data tool for data ingestion into the HDFS for storage. The suggested scheme has been assessed using MATLAB and compared to recent techniques based on different evaluation parameters. The results demonstrate that the CHROA method outperforms existing methods such as ABC, GSA, and WD-FA with a maximum average throughput of 70.122 kbps. Based on these statistical tests, it is clear that there is a significant variation in terms of the Average Throughput; hence, it was proved that CHROA is outperforming the other algorithms significantly.

This study suggests integrating IoE with Unmanned Aerial Vehicles (UAVs) to overcome network coverage and resource constraints, and designing routing protocols based on contextual information to enhance the network performance. In addition, using a load-balancing strategy supported by clustering can efficiently manage IoT activities while considering energy and performance requirements. The proposed dynamic clustering exhibits better performance. Although the proposed approach shows promising results, future research is required to develop AI-based performance enhancement strategies for the system’s future. Traffic shaping regulates the flow of network traffic to meet specific bandwidth or latency requirements, while traffic policing monitors and enforces traffic rate limits. These techniques are typically implemented in network devices such as routers or switches to control traffic flow.

HA proxies are commonly used to improve the system reliability and load balancing by distributing incoming network traffic across multiple backend servers. HA proxies help ensure high availability by handling traffic redirection and failover. Overall, this study highlights the potential of the CHROA method for energy-saving and load balancing in IoE environments and suggests several avenues for future research to enhance the network performance and efficiency, especially in the healthcare domain that requires low-latency real-time applications.

## Figures and Tables

**Table 2:** Review of literature on data retrieval methods for IoT.

Study	IoT Device (Node)	Communication Protocol	Power Consumption	Data Retrieval Method	Key Features
[43]	ESP8266	MQTT	0.37 W	HTTP GET	Low-cost, open-source platform
[38]	Raspberry Pi	MQTT	0.46 W	HTTP GET	Easy to program, powerful processing capabilities
[39]	CC3200	CoAP	0.65 W	CoAP GET/PUT	Secure communication using TLS/DTLS
[40]	ESP32	MQTT	0.73 W	MQTT	BLE connectivity, dual-core processor
[41]	Arduino MKR1000	HTTP	1.21 W	HTTP GET/POST	Low-power, onboard Wi-Fi
[42]	WSN430	6LoWPAN	0.42 mW	6LoWPAN GET/PUT	Low-power, long battery life

**Table 3:** Review of CHROA optimization algorithm applications.

Study	Objective	Optimization Target	Application	Results
[44]	Optimizing energy consumption	Low-power data retrieval systems in IoT	Temperature and humidity monitoring	Significant improvements in energy efficiency
[45]	Optimizing performance	WSN	Energy consumption, coverage, and connectivity	Significant improvement in energy efficiency
[46]	Optimizing routing	Low-power WSN	Energy consumption and reliable communication	Outperformed traditional routing protocols in energy efficiency

**Table 4:** Comparison of CHROA model with existing methods under different numbers of nodes.

Network Lifetime (Rounds)				
No. of Nodes	ABC	GSA	WD-FA	CHROA
100	12,739	15,101	17,995	18,972
200	13,316	15,364	18,893	20,069
300	15,538	17,047	19,323	21,419
400	17,688	19,410	21,567	23,874
500	20,225	21,265	23,193	24,404
**Total Energy Consumption (J)**				
**No. of Nodes**	**ABC**	**GSA**	**WD-FA**	**CHROA**
100	10.70	10.05	8.55	6.84
200	13.82	10.71	8.94	8.03
300	17.65	15.28	12.02	10.07
400	19.94	18.91	15.26	11.12
500	21.28	19.56	15.82	13.84
**Throughput (Kbps)**				
**No. of Nodes**	**ABC**	**GSA**	**WD-FA**	**CHROA**
100	39.04	40.28	52.75	61.36
200	46.45	47.65	57.35	66.96
300	51.24	51.72	61.23	69.59
400	54.21	56.19	65.75	75.27
500	60.36	60.48	67.85	77.43

**Table 5:** Results analysis of the CHROA model using current approaches at various node counts.

Normalized Overhead (%) (NO)				
No. of Nodes	ABC	GSA	WD-FA	CHROA
100	8.742	6.075	5.720	3.570
200	10.874	8.734	5.830	4.160
300	11.868	10.72	6.600	4.740
400	15.595	13.46	8.830	5.390
500	15.813	14.83	8.350	6.110
**End-to-End Delay (s)**				
**No. of Nodes**	**ABC**	**GSA**	**WD-FA**	**CHROA**
100	2.938	1.764	1.431	1.136
200	3.935	2.567	2.134	1.742
300	7.937	5.482	4.390	3.589
400	9.942	8.610	6.492	4.571
500	13.945	11.490	8.481	5.896

**Table 6:** Comparison of CHROA with existing techniques.

Performance Metrics	CHROA	ABC	GSA	WD-FA	Performance Metrics
Average TEC (mJ/bit)	0.1736	0.2059	0.1891	0.2031	Average TEC (mJ/bit)
Average Throughput (Kbps)	70.122	58.247	59.957	60.819	Average Throughput (Kbps)
ETE Delay (s)	0.0643	0.0781	0.0716	0.0752	ETE Delay (s)
Normalized Overhead	0.4069	0.4808	0.4437	0.4784	Normalized Overhead
Network Lifetime (s)	510.256	445.561	481.725	467.865	Network Lifetime (s)
Total Energy Consumption (Joule)	5.1289	6.7855	6.2234	6.7464	Total Energy Consumption (Joule)
Throughput (Kbps)	69.485	55.621	58.072	58.961	Throughput (Kbps)

**Table 7:** Variance (ANOVA) test on comparison of algorithms.

Performance Metric	F-Statistic	*p*-Value
Average TEC (mJ/bit)	0.157	0.854
Average Throughput (Kbps)	4.706	0.014
ETE Delay (s)	0.026	0.876
Normalized Overhead	0.066	0.8
Network Lifetime (s)	0.378	0.693
Total Energy Consumption (Joule)	0.722	0.531
Throughput (Kbps)	0.494	0.622

**Table 8:** Wilcoxon signed-rank test on comparison of algorithms.

Performance Metric	Z-Statistic	*p*-Value
Average TEC (mJ/bit)	−0.833	0.407
Average Throughput (Kbps)	2.485	0.013
ETE Delay (s)	−0.246	0.807
Normalized Overhead	−0.399	0.691
Network Lifetime (s)	0.618	0.539
Total Energy Consumption (Joule)	−1.334	0.182
Throughput (Kbps)	1.242	0.219

## Data Availability

Not Applicable.
